# Preliminary study on changes in BDNF in patients with depression after percutaneous coronary intervention

**DOI:** 10.1192/j.eurpsy.2023.232

**Published:** 2023-07-19

**Authors:** S. Medved, S. Sović, L. Ganoci, N. Božina, M. Šagud, J. Bulum, A. Mihaljević-Peleš

**Affiliations:** 1 Department of Psychiatry and Psychological Medicine, University Hospital Centre Zagreb; 2 Department of Medical Statistics, Epidemiology and Medical Informatics, “Andrija Štampar” School of Public Health; 3 Department of Laboratory Diagnostics, University Hospital Centre Zagreb; 4 School of Medicine University of Zagreb; 5 Department of Cardiovascular Diseases, University Hospital Centre Zagreb, Zagreb, Croatia

## Abstract

**Introduction:**

Depression and coronary artery disease (CAD) are often comorbid conditions. The presence of depression significantly interferes with the recovery after CAD therapy intervention, such as percutaneous coronary intervention (PCI), one of the most common medical procedures in developed countries. Brain derived neurotrophic factor (BDNF) has a major role in angiogenesis and neuromodulation. Its levels were previously shown to be reduced in patients with depression, and latest studies indicate similar in patients with CAD. However, the correlation of BDNF levels and depression after CAD treatment is unknown.

**Objectives:**

The aim of this preliminary study is to assess the changes in BDNF levels in patients with depressive symptoms during a six-month period upon PCI.

**Methods:**

Antidepressant-free participants that underwent PCI with stent placement due to myocardial infarction or angina pectoris were enrolled in the study. Depressive symptoms were evaluated at baseline using the Beck’s Depression Inventory II (BDI-II) with a cut-off score ≥20 indicating moderate depression. Serum BDNF levels were measured from blood samples drawn a day after (baseline) and six-months upon a successful PCI without complications. The t-test for dependent samples was used with marked significant differences at p<0,05.

**Results:**

Altogether, 76 participants were included in the study, of which 25 finished a six-month follow-up. Participants with BDI-II≥20 at baseline had higher serum BDNF levels in the second measurement (M=23,12, SD=6,20; M=32,02, SD=12,26, respectively). No significant difference was found in serum BDNF levels in measurements between participants with and without depressive symptoms (t=0,33, p=0,74; t=-1,40, p=0,18, respectively). Statistically significant difference was found between serum BDNF in the first and second measurement in the overall sample (t=-2,28, p=0,03) and in participants with baseline moderate depressive symptoms (t=-2,46, p= 0,03), but not in those without (t=-0,59, p=0,57).

**Conclusions:**

Serum BDNF levels in participants with baseline moderate depressive symptoms increased after a six-month period upon successful PCI treatment, whereas that trend was not observed in participants without depressive symptoms. This highlights the potential synergistic role of BDNF in comorbid depression and CAD.

**Disclosure of Interest:**

None Declared

